# Electroacupuncture alleviates osteoarthritis and is associated with changes in gut microbiota and steroid biosynthesis-related metabolites

**DOI:** 10.3389/fmolb.2026.1769807

**Published:** 2026-08-10

**Authors:** Junjie Ma, Lingxing Ouyang, Xuan Zhou, Xiaoyun Peng, Mei Zhao, Huimeng Zhu, Bingyao Huang, Jun Yan

**Affiliations:** 1 Rehabilitation Medicine Department, Shenzhen Hospital of Integrated Traditional Chinese and Western Medicine, Shenzhen, China; 2 Acupuncture and moxibustion Rehabilitation Department, Heyuan Traditional Chinese Medicine Hospital, Heyuan, China

**Keywords:** electroacupuncture, gut microbiota, metabolism, osteoarthritis, steroid biosynthesis

## Abstract

**Background:**

Electroacupuncture (EA) is widely used for osteoarthritis (OA), but its mechanisms remain unclear. This study investigated the therapeutic effects of EA on OA in rats and its potential mechanisms.

**Methods:**

A rat model of knee OA was established via intra-articular injection of sodium monoiodoacetate. The effects of EA on behavioral indices (paw withdrawal threshold (PWT), body weight, knee width), cartilage pathology (HE staining, Safranin O-fast green staining, TUNEL), and inflammatory factors (IL-6, IL-1β, TNF-α) were assessed. Gut microbiota and metabolites were analyzed using 16S rRNA sequencing and metabolomics. Finally, integrative analysis of differential gut microbiotas and metabolites was conducted.

**Results:**

EA alleviated joint pain, increased PWT, reduced knee swelling, promoted cartilage repair, and inhibited chondrocyte apoptosis in OA rats. Additionally, EA decreased IL-6, IL-1β, and TNF-α levels in both serum and synovial fluid. EA altered gut microbiota composition, increasing *Muribaculaceae* and *Lachnospiraceae* at the family level, and *Clostridia* and *Campylobacteria* at the class level, while decreasing *Alphaproteobacteria* and *Actinobacteria*. Functional analysis showed changes in pathways like Biosynthesis of vancomycin group antibiotics, Folate biosynthesis, and Vitamin B6 metabolism. Metabolomics revealed that EA modulated metabolic pathways in OA rats, particularly the Steroid biosynthesis pathway. Specifically, 7-Dehydrocholesterol was decreased in the OA group compared to the Control group, while EA treatment increased its levels. Integrated analysis revealed associations between 41 gut microbiotas and 47 metabolites.

**Conclusion:**

EA alleviated OA and was associated with changes in gut microbiota composition and steroid biosynthesis-related metabolites. These findings suggest that the gut microbiota–metabolite axis may be involved in the effects of EA on OA.

## Introduction

Osteoarthritis (OA) is a heterogeneous whole-joint disease that is increasingly prevalent due primarily to aging and obesity, representing a major disease burden worldwide (Kloppenburg et al., 2025). Currently, with the continuous acceleration of global aging process, the prevalence of OA is steadily increasing (He et al., 2024). Its main characteristics include cartilage damage, synovial inflammation, subchondral bone sclerosis, marginal bone loss, and osteophyte development, which severely reduce patients’ quality of life (Xi et al., 2025). The development of OA is multifactorial, encompassing elements like mechanical strain, inflammatory reactions, imbalances in cartilage metabolism, and alterations in gut microbiota (Cao et al., 2024; Guan et al., 2022). Traditional treatment methods include pharmacological therapy (such as nonsteroidal anti-inflammatory drugs), physical therapy, and surgical intervention, but these approaches often only alleviate symptoms and fail to fundamentally halt disease progression (Siddiq et al., 2024). Therefore, the search for a safe and effective treatment method holds significant clinical importance.

As a key element of traditional Chinese medicine, acupuncture boasts an extensive history and profound clinical practice in managing OA (Wang et al., 2020). Research has demonstrated its efficacy in substantially alleviating pain in OA patients, optimizing joint mobility, and elevating overall quality of life (Yin et al., 2023). However, the precise mechanisms behind acupuncture remain not fully understood, which to some extent limits its widespread application and further development in the treatment of OA. Electroacupuncture (EA) is an advanced acupuncture technique that amplifies its therapeutic potential by introducing a mild electric current to the stimulated acupuncture points (Erasmus et al., 2024). Previous studies have indicated that EA can produce anti-inflammatory, pain-relieving, and tissue-regenerating effects by modulating the neuro-immune-endocrine network (Wan et al., 2025; Xu et al., 2020). However, the specific mechanisms of EA in treating OA remain unclear.

In recent years, as emerging technologies like microbiomics and metabolomics have advanced rapidly, more and more research has begun to explore the connections between gut microbiota and health or disease (Midya et al., 2024). The gut microbiota, often referred to as the “second genome” of the human body, plays a crucial role in numerous vital physiological functions such as nutrient metabolism, immune regulation, and maintaining barrier integrity (Prosty et al., 2024; Chandrasekaran et al., 2024). Research has shown that imbalances in the gut microbiota are strongly linked to the onset and progression of various diseases, with OA being no exception (Yu et al., 2025). In patients with OA, notable alterations in the composition and diversity of the gut microbiota have been observed, and these changes are correlated with disease severity and clinical manifestations (Gaspar et al., 2025). Meanwhile, metabolomics can comprehensively analyze small-molecule metabolites within biological systems, reflecting the metabolic status and physiological and pathological changes of the body (Wishart, 2019). Through metabolomics research on OA patients and animal models, it has been found that OA development is accompanied by a series of metabolic pathway disorders, such as energy metabolism, lipid metabolism, and amino acid metabolism (Wang et al., 2025). Therefore, a detailed investigation into how acupuncture influences gut microbiota and metabolomics in OA rats may reveal new mechanisms of acupuncture in treating OA and provide new ideas and targets for OA treatment.

Based on this, the present study aims to systematically evaluate the therapeutic effects of EA on OA rats. By exploring its potential mechanisms from multiple dimensions, including pain relief, cartilage protection, inflammation inhibition, gut microbiota regulation, and metabolomics changes, this study seeks to offer a more robust scientific foundation and therapeutic targets for treating OA with EA.

## Materials and methods

### Animals

Male Sprague-Dawley rats, weighing 200–250 g, were obtained from Guangzhou Ruige Biotechnology Co., Ltd. The rats were randomly assigned into three groups, with 6 rats in each group: Control, OA, and OA + EA groups. Prior to the experiment, the rats were allowed to acclimate for 1 week in a controlled environment maintained at a temperature of (22 ± 2) °C and a relative humidity of (50 ± 10) %, with unrestricted access to food and water. The OA model was established via intra-articular injection of 50 μL of 60 mg/mL sodium monoiodoacetate (i.e., 3 mg) (Philpott and McDougall, 2020). Rats in the Control group received an intra-articular injection of the same volume of saline. Rats in the OA + EA group underwent EA treatment starting on the second day after modeling, with 5 sessions per week, each lasting 20 min, for a total of 3 weeks. The acupuncture points used were: ST36 (Zusanli, 0 mm), EX-LE5 (Neixiyan, 5 mm), and Heting (2 mm). These acupoints were selected based on traditional Chinese medicine theory. ST36 was used as a distal point, whereas EX-LE5 (Neixiyan) and Heting were selected as local points around the knee to relieve pain, swelling, and restricted joint movement. The acupuncture method was as follows: Rats were gently restrained in a fixation device to limit excessive movement during EA treatment. Acupuncture points were located according to the “Acupuncture Points Atlas for Laboratory Animals.” Disposable acupuncture needles (0.25 mm × 13 mm) were inserted unilaterally to a depth of 5 mm, and electrodes were attached to the needle handles. The points were connected to a SDZ-II electronic acupuncture instrument, which delivered a continuous sparse wave stimulation at a frequency of 2 Hz and an intensity of 1 mA for 20 min (Zhang et al., 2023). Behavioral changes and pain responses in the rats were observed once a week, and body weight and knee width were measured. The rats were anesthetized and placed in a supine position on an operating pad, and knee width was measured using a vernier caliper (Pu et al., 2026). After tissue collection, macroscopic photographs were taken to observe the color changes in the cartilage surface, the size of cartilage defects, and the degree of erosion. Blood, synovial fluid, and fecal samples were collected.

### Paw withdrawal threshold (PWT) detection

Pain behavior was evaluated with von Frey filaments. Rats were positioned in a specialized cage equipped with a metal mesh floor to access the plantar surface of the hind paws. After a 10-min acclimation period during which exploratory behavior subsided, von Frey filaments were applied. Each filament was pressed perpendicularly against the plantar surface of the ipsilateral hind paw (excluding the footpad) until it bent, and held for 3 s. If a positive response was observed (i.e., paw withdrawal, shaking, or licking), a filament with lower force was used next. If no response was observed, the next filament of higher force was used, up to a cutoff bending force of 25 g (Philpott and McDougall, 2020; Li et al., 2020). PWT was evaluated on postoperative days 7, 14, and 21.

### Hematoxylin eosin (HE) staining

Decalcified sections of rat knee joints were stained with HE to observe changes in chondrocytes. Rat knee joint cartilage tissues were fixed using a tissue fixative (G1101, Servicebio). Sections were baked at 60 °C for 1–2 h, dewaxed to water, and stained with hematoxylin (G1003, Servicebio) for 5–10 min. They were then counterstained in PBS to enhance contrast. Following this, sections were stained with eosin for 3–5 min. Dehydration was performed using graded ethanol (95%–100%) for 5 min at each concentration. Afterward, sections were placed in xylene for 10 min, twice, and then mounted with neutral gum. The slides were examined under a fluorescence microscope (BA210T, Motic).

### Safranin O-fast green staining

To examine alterations in chondrocytes, rat knee joint sections were decalcified and stained with Safranin O-fast green. Cartilage tissues were embedded in paraffin and sectioned at a thickness of 2 μm. Sections were baked at 60 °C for 12 h. After dewaxing to water, the sections were stained with fast green, followed by Safranin O staining, and rinsed thoroughly with distilled water. Sections were dried using a hairdryer and then placed in xylene. After mounting with neutral gum, the slides were examined and photographed under a microscope.

### TdT-mediated dUTP nick-end labeling (TUNEL)

Apoptosis in knee joint cartilage tissues was assessed with a TUNEL assay kit (G1502, Servicebio). Cartilage tissue sections were baked at 60 °C for 30–60 min, then dewaxed to water. Each section was treated with 100 μL of Proteinase K (G1205, Servicebio) and incubated at 37 °C for 20 min. Tissues were saturated with an adequate volume of membrane-breaking solution (G1204, Servicebio) and incubated for 20 min at room temperature. Following the membrane-breaking process, samples were washed with PBS in the same manner. Each sample was covered with 100 μL 1× Equilibration Buffer over the entire area and incubated for 10–30 min at room temperature. After preparing the TdT enzyme incubation buffer, the majority of 1× Equilibration Buffer was blotted away from the treated area using absorbent paper. Subsequently, 50 μL of TdT enzyme incubation buffer was applied to the tissue and incubated for 60 min at 37 °C in the dark. Nuclei were stained with DAPI (G1012, Servicebio) by incubating sections at 37 °C for 10 min. Sections were then mounted with neutral gum and examined under a fluorescence microscope.

### Enzyme linked immunosorbent assay (ELISA)

The concentrations of inflammatory cytokines IL-6, TNF-α, and IL-1β in serum and synovial fluid were measured using ELISA. ELISA kits for IL-6 (RX302856R), TNF-α (RX302058R), and IL-1β (RX302869R), all from RUIXIN BIOTECH, were utilized to determine the levels of these cytokines following the manufacturer’s protocols.

### 16S rRNA sequencing

Initially, fecal samples were processed to obtain total DNA. The extracted DNA was then used as a template to amplify the V3-V4 regions of the 16S rRNA gene, utilizing universal primers 341F (5′–CCTAYGGGRBGCASCAG–3′) and 806R (5′–GGACTACNNGGGTATCTAAT–3′). 16S rRNA sequencing was performed using Illumina NovaSeq PE250 to obtain raw sequencing data for subsequent information analysis. Species information was assigned to the representative sequences of each operational taxonomic unit, and the abundance distribution based on species was obtained. The α-diversity indices (Shannon and Simpson) of each sample were calculated using QIIME 2 software. Partial Least Squares Discriminant Analysis (PLS-DA) was utilized to construct a model that links microbial composition with sample classification. The Kruskal–Wallis rank-sum test was employed to identify species and functions with significant abundance differences among different groups. Linear Discriminant Analysis Effect Size (LEfSe) software was utilized to examine differential microbiota, identify gut microbiotas with significant differences between groups, and explore their biological functions in each group.

### Metabolomics

As previously described, liquid chromatography-mass spectrometry analysis was initially conducted on the samples (Zhu D. et al., 2024). The raw mass spectrometry data files were then converted to mzXML format with MSConvert tool from the Proteowizard software package (v3.0.8789) (Rasmussen et al., 2022). Subsequently, peak detection, filtering, and alignment were performed with the R XCMS software package (v3.12.0) to obtain a quantitative list of metabolites (Domingo-Almenara and Siuzdak, 2020). Systematic errors were eliminated through support vector regression correction using quality control (QC) samples. Subsequently, metabolites with a coefficient of variation below 30% in QC samples were selected for further analysis (Le Pogam et al., 2019). Metabolite identification was performed by searching against spectral databases like HMDB, MassBank, LipidMaps, mzCloud, KEGG, and an in-house metabolite standard database (library search). The R Pheatmap package was utilized to scale quantitative matrix data of differential metabolites and to perform hierarchical clustering of both samples and metabolites, generating a clustered heatmap. Volcano plots intuitively illustrated the distribution and trends of differential metabolites between the two sample groups. Venn diagrams were employed to determine the number of overlapping and distinct differential metabolites across various comparison groups. Kyoto Encyclopedia of Genes and Genomes (KEGG) pathway enrichment analysis of differential metabolites was carried out with MetaboAnalyst (www.metaboanalyst.ca) (Pang et al., 2024). Box plots were used to illustrate the overall distribution of a dataset, highlighting the characteristics of the data distribution.

### Statistical analysis

For statistical analysis, GraphPad Prism 8.0 software was utilized. Quantitative data were presented as mean ± standard deviation. Comparisons among multiple groups were conducted using one-way analysis of variance (ANOVA). *P* < 0.05 was considered to indicate a statistically significant difference.

## Results

### EA alleviated joint pain in rats with OA

**FIGURE 1:**
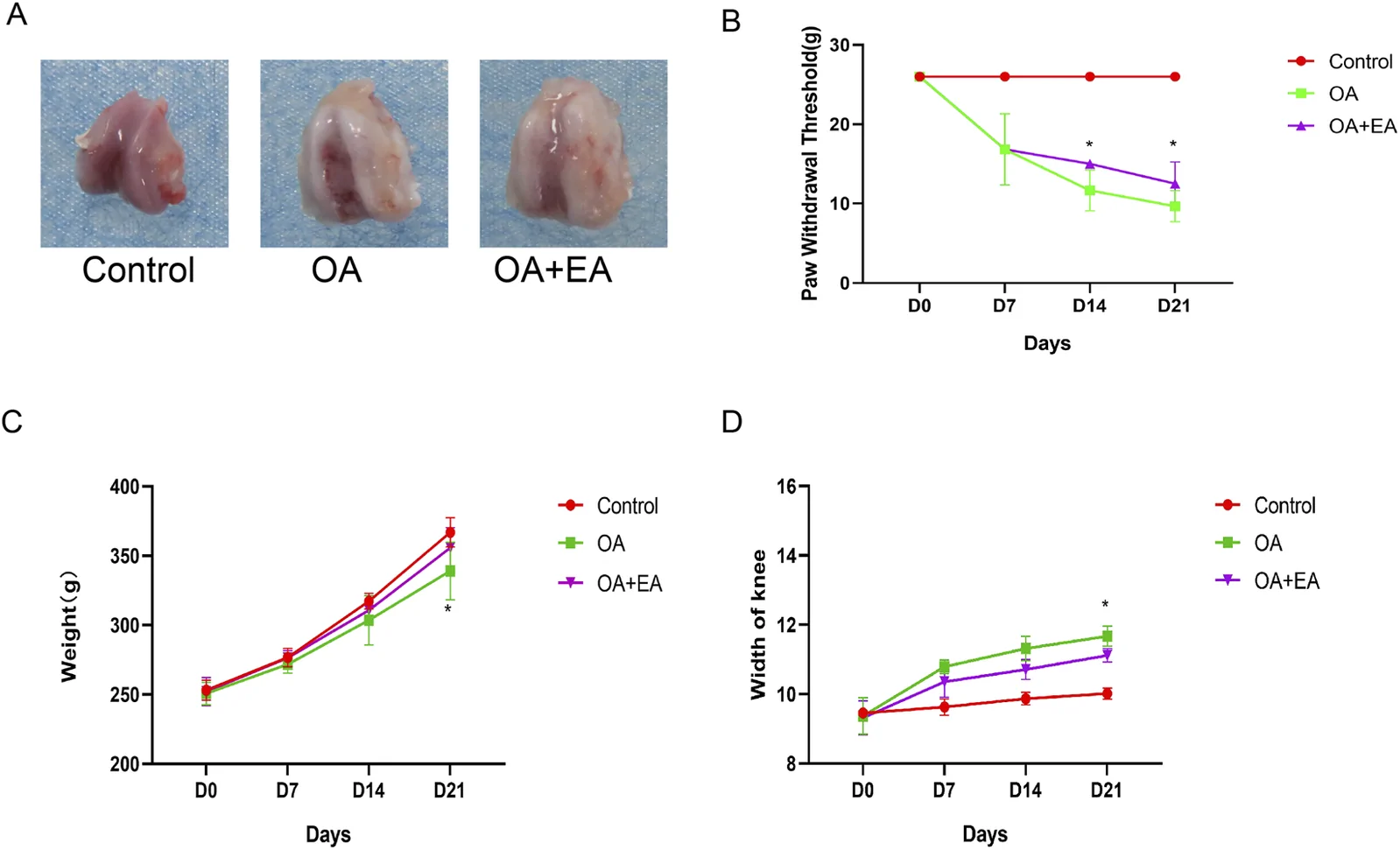
EA alleviated joint pain in rats with OA. **(A)** Macroscopic comparison of knee joint cartilage in rats at the time of tissue collection. **(B)** Detection of PWT. **(C)** Changes in body weight of rats throughout the experimental period. **(D)** Changes in knee joint width in rats during the experimental period. *P < 0.05.

Initially, we established the rat OA model and applied EA treatment. Upon tissue collection, a macroscopic comparison of the knee joint cartilage revealed that, compared to the Control group, the cartilage surface in the OA group exhibited unevenness and noticeable damage. After EA treatment, partial recovery of the cartilage damage on the knee joint surface was observed (Figure 1A). In the OA group, the PWT was markedly reduced from Day 7 to Day 21 when contrasted with the Control group. After EA treatment, the PWT of the rats increased (Figure 1B). Additionally, compared to the Control group, the body weight of rats in the OA group gradually decreased, while the knee joint width gradually increased from Day 7 to Day 21. After EA treatment, the body weight of rats increased, and knee joint width decreased (Figures 1C,D).

### EA ameliorated cartilage destruction in OA rat

**FIGURE 2:**
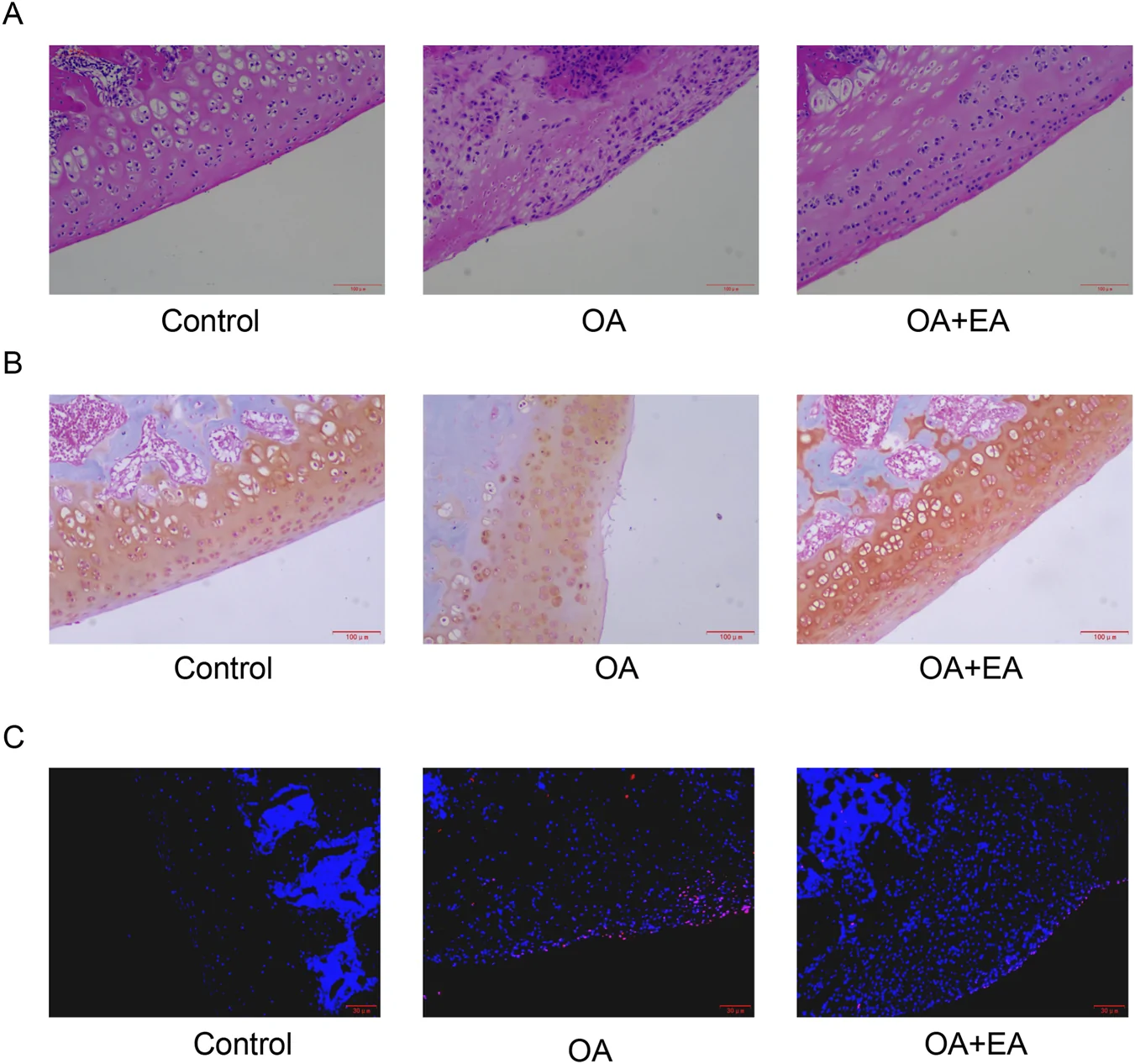
EA ameliorated cartilage destruction in OA rat. **(A)** HE staining showing changes in knee joint cartilage. Scale bar = 100 μm. **(B)** Safranin O-fast green staining showing changes in knee joint cartilage. Scale bar = 100 μm. **(C)** TUNEL staining of apoptosis in knee joint cartilage tissues. Scale bar = 30 μm.

Next, we analyzed the pathological conditions of the knee joint cartilage tissues in rats. Histological analysis via HE staining showed that the cartilage in the OA group was thinner and more irregular compared to the Control group. After EA treatment, the cartilage became thicker and more regular (Figure 2A). Safranin O-fast green staining revealed cartilage erosion in the OA group, which was significantly improved after EA intervention (Figure 2B). Additionally, TUNEL staining indicated that the OA group had a higher rate of cell apoptosis in knee joint cartilage and surrounding tissues compared to the Control group. EA treatment alleviated apoptosis caused by OA (Figure 2C).

### EA alleviated inflammation in OA rat

**FIGURE 3:**
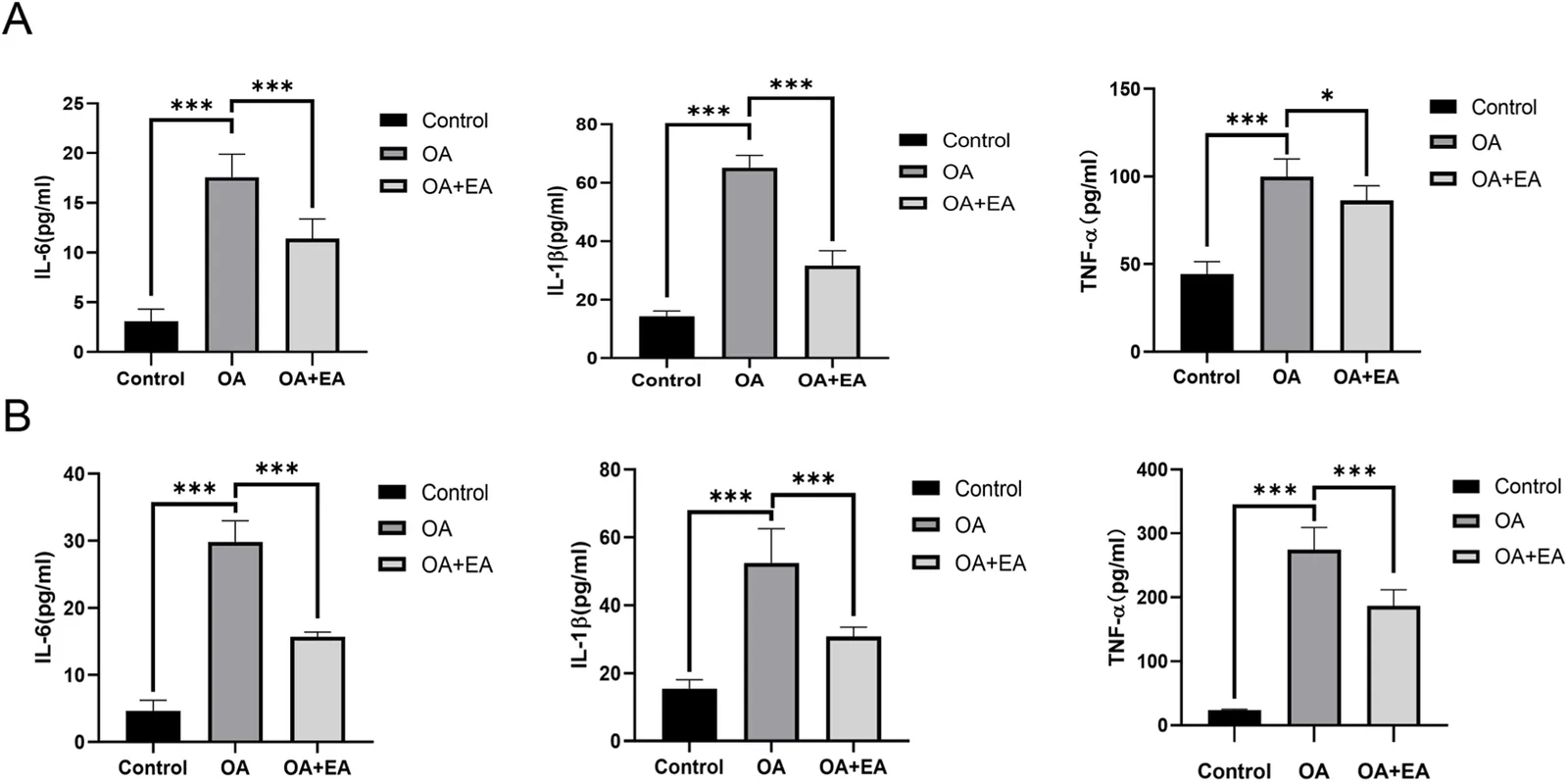
EA alleviated inflammation in OA rats. **(A,B)** ELISA detection of inflammatory cytokines IL-6, IL-1β, and TNF-α levels in serum and synovial fluid. *P < 0.05, ***P < 0.001.

Additionally, the concentrations of inflammatory cytokines IL-6, IL-1β, and TNF-α in rat serum and synovial fluid were measured. Compared with the Control group, these cytokine levels were markedly increased in the OA group. After EA treatment, these cytokine levels were reduced (Figures 3A,B).

### EA regulated gut microbiota dysbiosis in rats with OA

**FIGURE 4:**
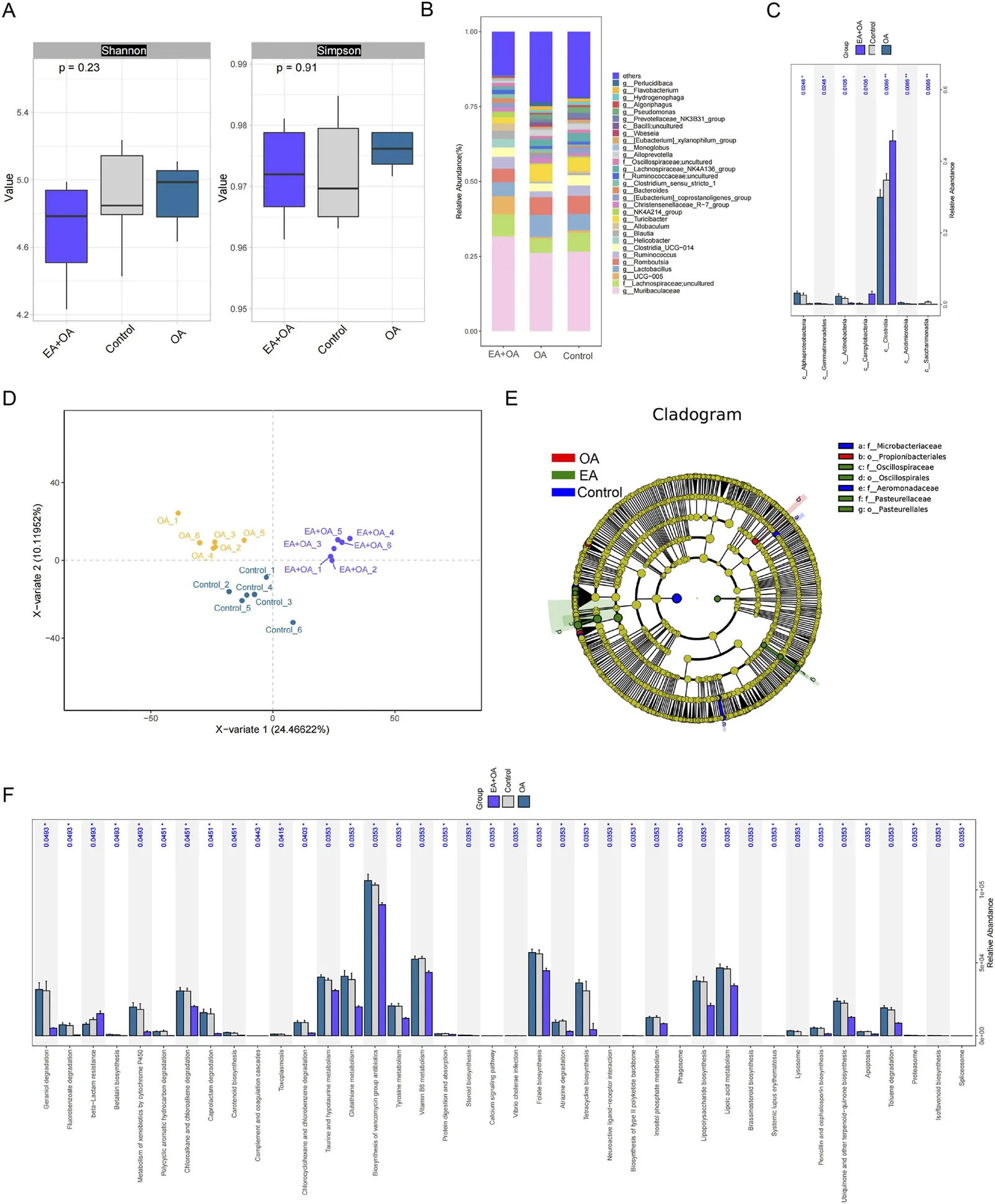
EA regulated gut microbiota dysbiosis in rats with OA. **(A)** Shannon and Simpson indices demonstrated α-diversity. **(B)** Bar plot showing species information with relative abundance above 1% at the family level. **(C)** Kruskal–Wallis statistical analysis of species at the class level. **(D)** β-diversity: PLS-DA established a relationship model between microbial composition and sample categories. **(E)** Differential analysis using LEfSe software. **(F)** Functional analysis of differential gut microbiotas (Kruskal–Wallis statistical analysis).

Next, we performed 16S rRNA sequencing to analyze the differential gut microbiotas among groups. Shannon and Simpson indices were employed to demonstrate α-diversity. The findings indicated that EA did not significantly affect the diversity of gut microbiota (Figure 4A), suggesting that EA may not affect OA through the regulation of α-diversity. The bar plot results displayed species information with relative abundance above 1% at the family level. *Muribaculaceae* had the highest abundance in the Control, OA, and EA groups, all exceeding 25%, and it was significantly increased in the EA group. Additionally, compared to the Control group, the abundance of *Lachnospiraceae* decreased in the OA group and was restored after EA treatment (Figure 4B). The Kruskal–Wallis statistical analysis of species at the class level showed that EA significantly upregulated the abundance of *Clostridia* and *Campylobacteria* while downregulating *Alphaproteobacteria* and *Actinobacteria* (Figure 4C). Moreover, PLS-DA revealed significant differences among the groups (Figure 4D). Subsequently, LEfSe was utilized for differential analysis. The results revealed the key gut microbiota in the OA group were *Propionibacteria*, while the key microbiota in the EA group were *Oscillospiraceae*, *Oscillospirales*, *Pasteurellaceae*, and *Pasteurellales* (Figure 4E). Furthermore, functional analysis of the differential gut microbiotas showed significant changes in pathways such as Biosynthesis of vancomycin group antibiotics, Folate biosynthesis, and Vitamin B6 metabolism (Figure 4F).

### Metabolomics analysis of metabolic changes in OA rats

**FIGURE 5:**
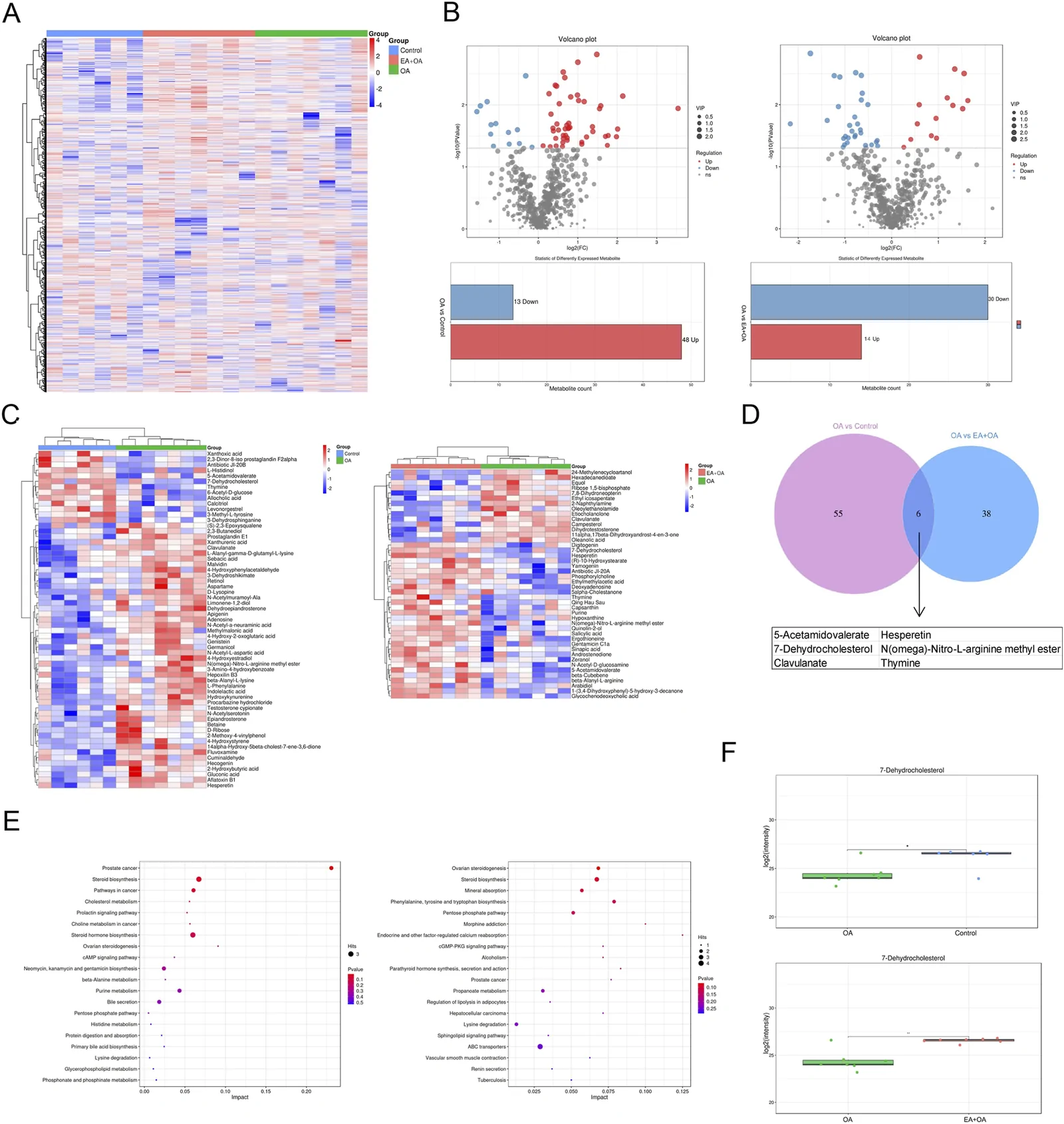
Metabolomics analysis of metabolic changes in OA rats. **(A)** Heatmap displayed metabolites in samples from Control, OA, and EA groups. **(B)** Volcano plot showing differential metabolites between OA vs. Control group and OA vs. EA group. **(C)** Heatmap displayed specific upregulated and downregulated metabolites in the OA vs. Control group and the OA vs. EA group. **(D)** Venn diagram showed common differential metabolites. **(E)** KEGG pathway enrichment analysis of differential metabolites. **(F)** Analysis of metabolites in the common differential pathway, Steroid biosynthesis. *P < 0.05.

Next, we used metabolomics to analyze the metabolic changes in rats across different groups. First, the clustered heatmap displayed the metabolites in samples from the Control, OA, and EA groups (Figure 5A). Volcano plots intuitively showed the distribution and trends of differential metabolites between the OA vs. Control group and OA vs. the EA group. There were 48 upregulated and 13 downregulated differential metabolites in the OA vs. Control group. In the OA vs. EA group, there were 14 upregulated and 30 downregulated differential metabolites (Figure 5B). Further, heatmaps displayed the specific upregulated and downregulated metabolites in the OA vs. Control group and the OA vs. EA group (Figure 5C). Venn diagram of differential metabolites showed the common differential metabolites between the OA vs. Control group and the OA vs. EA group. The results indicated that 6 common differential metabolites were shared, including 5-Acetamidovalerate, Hesperetin, 7-Dehydrocholesterol, N (omega)-Nitro-L-arginine methyl ester, Clavulanate, and Thymine (Figure 5D). Subsequently, KEGG pathway enrichment analysis was conducted on differential metabolites. The results revealed that in OA vs. Control group, metabolites were predominantly enriched in pathways like Steroid biosynthesis, Purine metabolism, and Bile secretion. In contrast, in the OA vs. EA group, metabolites were mainly enriched in pathways like Steroid biosynthesis, ABC transporters, and Mineral absorption (Figure 5E). Finally, analysis of metabolites in the common differential pathway, Steroid biosynthesis, revealed that compared to the Control group, 7-Dehydrocholesterol level was decreased in the OA group. After EA treatment, the 7-Dehydrocholesterol level increased (Figure 5F).

### The integrated analysis of differential gut microbiotas and metabolites

**FIGURE 6:**
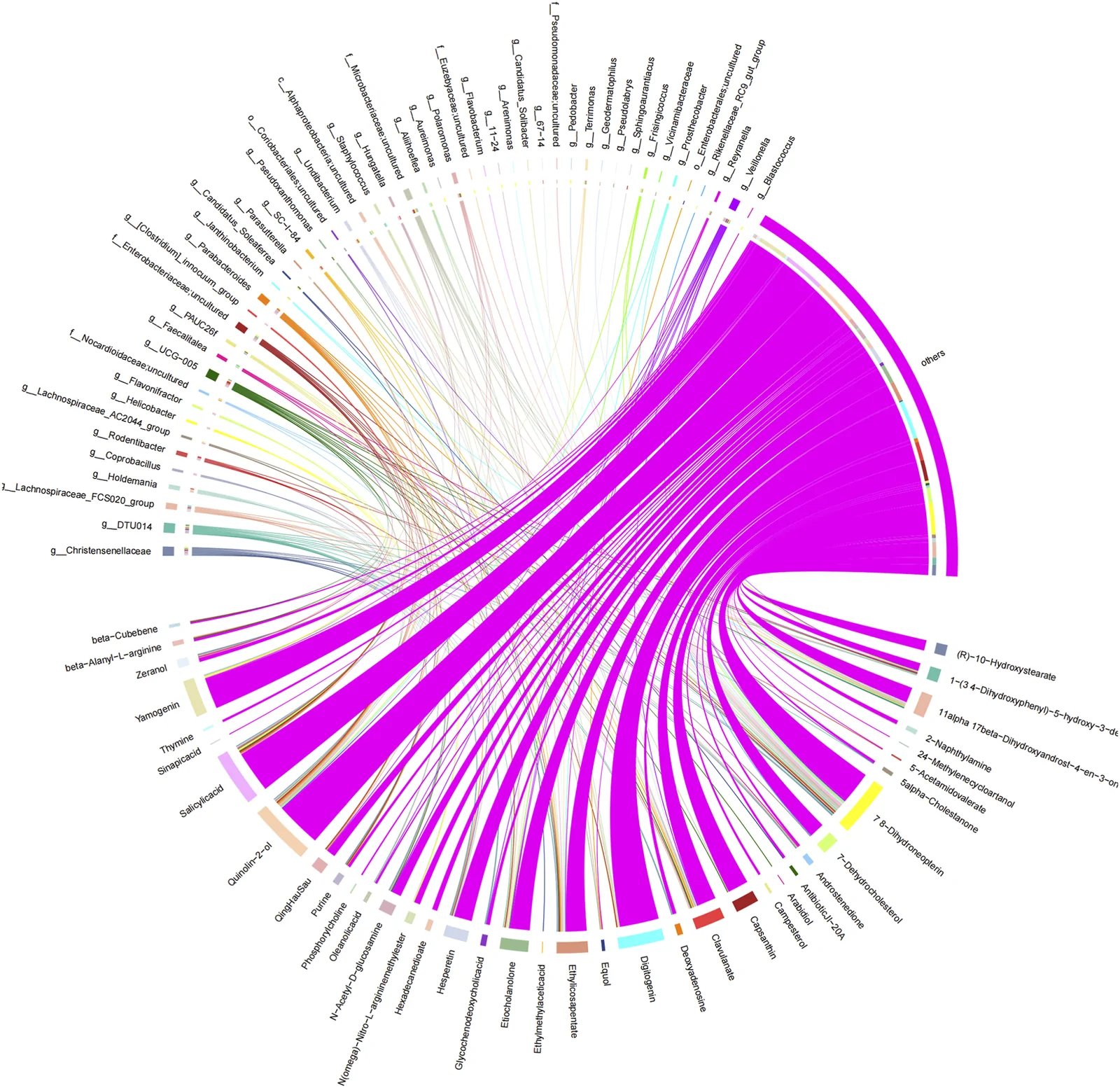
Integrated analysis of differential gut microbial taxa and metabolites. Chord diagram showing the associations between differential gut microbial taxa and metabolites in the OA vs. EA comparison.

Through the integrated analysis of differential gut microbiotas and metabolites in the OA vs. EA group, the chord diagram results showed that 41 types of gut microbiotas and 47 metabolites were interconnected. Among them, metabolites such as Quinolin-2-ol, Etiocholanolone, and Digitogenin had multiple gut microbiotas associations. In the Steroid biosynthesis pathway, 7-Dehydrocholesterol was associated with 28 types of gut microbiotas, among which the highest correlations were found with *Janthinobacterium*, *Aeromonas*, and *Microtrichales* (Figure 6).

## Discussion

This study explored the therapeutic effects of EA on OA from multiple dimensions, including pain relief, cartilage protection, inflammation inhibition, gut microbiota regulation, and metabolomic changes. Our results showed that EA alleviated OA and was associated with changes in gut microbiota abundance and steroid biosynthesis-related metabolites. These findings suggest that the “gut microbiota–metabolite–joint axis” may be involved in the effects of EA on OA. However, the present data support an association rather than a direct causal relationship.

Traditionally, acupuncture is believed to primarily work by stimulating acupoints to regulate the flow of Qi and blood in meridians, thereby achieving effects such as unblocking meridians, harmonizing Qi and blood, and relieving pain (Wu et al., 2020). However, from the perspective of modern medicine, its mechanisms of action may involve multiple complex physiological and pathological processes, including neural regulation, immune modulation, and inflammation inhibition (Chen et al., 2024). For example, acupuncture may exert analgesic effects by regulating the release of neurotransmitters including endorphins and serotonin (Trinh et al., 2022). Additionally, acupuncture can inhibit inflammatory responses by modulating the activity of immune cells and the secretion of cytokines (Wang et al., 2023). These studies highlight the important role of acupuncture in treating diseases. In this study, the established rat OA model exhibited typical joint pathological features, laying the foundation for exploring the therapeutic mechanisms of EA. We found that EA treatment significantly improved pain behaviors in rats with OA, as evidenced by increased PWT. This result indicated that EA could effectively alleviate mechanical pain sensitivity caused by OA, suggesting that it may relieve pain through the regulation of neural pathways or local inflammatory responses. Additionally, EA also improved the weight loss and increased knee joint width in OA rats, further demonstrating its positive impact on overall health status. Some studies have indicated that EA can activate the expression of certain growth factors like insulin-like growth factor, which can promote chondrocyte proliferation and extracellular matrix synthesis, thereby repairing damaged cartilage (Wang et al., 2022; Wen et al., 2021). Further histological analysis showed that EA significantly improved pathological changes in the knee joint cartilage of OA rats, including cartilage thickening, reduced cartilage erosion, and decreased apoptosis. These findings suggested that EA not only alleviated physical cartilage damage but may also delay cartilage degeneration by inhibiting apoptosis. This discovery provides important evidence for the potential application of EA as a chondroprotective agent, indicating that it may function by regulating extracellular matrix metabolism and signaling pathways in the chondrocyte microenvironment.

Inflammatory responses are crucial in OA progression, with inflammatory cytokines like IL-6, IL-1β, and TNF-α being important mediators that trigger joint inflammation and cartilage destruction (De Roover et al., 2023). Research has shown that EA can modulate the function of immune cells, such as inhibiting the polarization of macrophages towards a pro-inflammatory phenotype and reducing secretion of inflammatory cytokines (Wang et al., 2021). Additionally, EA can also inhibit the gene transcription of inflammatory cytokines by regulating inflammatory pathways like NF-κB (Liu et al., 2020). In this study, rats in the OA group exhibited significantly higher levels of inflammatory cytokines IL-6, IL-1β, and TNF-α in both serum and synovial fluid, which were notably decreased following EA treatment. This suggested that EA can effectively suppress inflammatory responses, likely by inhibiting the activation of inflammatory cells and reducing the secretion of inflammatory cytokines. This result further supports the potential of EA as a non-pharmacological anti-inflammatory modality.

In recent years, the role of gut microbiota in the development and progression of various diseases has attracted increasing attention, and OA is no exception (Yu et al., 2025). Imbalances in gut microbiota could influence the body’s immune function, metabolic status, and inflammatory responses, thereby contributing to the pathological process of OA (Basak et al., 2023). In this study, 16S rRNA sequencing revealed that EA did not significantly affect α-diversity of gut microbiota but changed its composition. At the family level, the abundance of *Muribaculaceae* was significantly higher in the EA group. *Muribaculaceae* is known to be involved in the production of short-chain fatty acids (SCFAs), which possess anti-inflammatory, immune-regulating, and gut barrier-protecting properties (Zhu Y. et al., 2024). Therefore, *Muribaculaceae* may influence joint inflammation and cartilage metabolism via the bloodstream, thereby improving OA symptoms. *Lachnospiraceae*, which is involved in polysaccharide degradation and SCFA production and is crucial for maintaining gut microbiota balance (Sun et al., 2021; Zaplana et al., 2023), decreased in the OA group but was restored after EA treatment. The recovery of *Lachnospiraceae* may help improve the gut environment in OA rats. At the class level, EA significantly increased the abundance of *Clostridia* and *Campylobacteria* while decreasing that of *Alphaproteobacteria* and *Actinobacteria*. *Clostridia* includes various butyrate-producing bacteria, whose metabolites can exert anti-inflammatory effects by regulating the Treg/Th17 balance (Li T. et al., 2024). *Actinobacteria* has been reported to be increased in OA-associated dysbiosis and may contribute to low-grade inflammation. Therefore, the decrease in *Actinobacteria* after EA treatment may be related to an improved inflammatory state (de Sire et al., 2025). These changes may be related to EA’s ability to improve systemic inflammation by modulating the gut microbiota. LEfSe analysis further indicated that *Propionibacteria* were significantly enriched in the OA group, whereas in the EA group, microbiota such as *Oscillospiraceae* were more prominent. Functional analysis of the differentially regulated microbiota by EA indicated that these microbiotas affected pathways such as Biosynthesis of vancomycin group antibiotics**,** Folate biosynthesis, and Vitamin B6 metabolism**.** Vitamin B6 metabolism is related to inflammatory responses (Zhu et al., 2023), and alterations in its metabolic pathways may be involved in EA’s regulation of OA inflammation. In summary, EA may selectively enrich anti-inflammatory microbiota and inhibit pro-inflammatory microbiota to restore gut microbiota balance, thereby alleviating OA through the ‘‘microbiota–immune’’ axis.

The development of metabolomics technology has provided a powerful tool for studying metabolic changes in diseases. By analyzing changes in metabolites within biological systems, metabolomics can offer deep insights into disease pathogenesis and the effects of therapeutic interventions (Vo and Trinh, 2024). In this study, metabolomics analysis revealed changes in metabolites in OA rats and the modulatory effects of EA. The OA vs. Control group and the OA vs. EA group exhibited distinct distributions of differential metabolites. KEGG pathway enrichment analysis showed significant changes in the Steroid biosynthesis pathway in both groups. Steroid biosynthesis has been reported to be closely related to OA (Martin et al., 2024). The levels of 7-Dehydrocholesterol in the Steroid biosynthesis pathway significantly increased after EA treatment, suggesting that EA might exert its anti-inflammatory and chondroprotective effects by modulating steroid metabolism. 7-Dehydrocholesterol is a precursor in cholesterol synthesis, and cholesterol plays a vital role in maintaining cell membrane integrity and cellular function (Li Y. et al., 2024; Keller et al., 2004). Abnormalities in its synthesis may lead to chondrocyte dysfunction, and EA’s regulation of this metabolite may help restore normal chondrocyte function. In addition, the common differential metabolite Hesperetin, a natural flavonoid compound with antioxidant and cyclooxygenase-2 inhibitory properties (Song et al., 2024), shows changes in levels that suggest EA may exert protective effects by modulating microbiota-host co-metabolites in a coordinated manner. Metabolites in the OA vs. Control group were also enriched in pathways like Purine metabolism and Bile secretion, while the OA vs. EA group exhibited significant changes in pathways like ABC transporters and Mineral absorption. These pathway alterations reflected the metabolic and energetic disturbances in OA rats, as well as EA’s regulatory effects. Furthermore, integrated analysis of differential microbiota and metabolites in the OA vs. EA group revealed connections between 41 types of gut microbiotas and 47 metabolites. Among them, metabolites such as Quinolin-2-ol, Etiocholanolone, and Digitogenin were closely associated with multiple gut microbiotas. In the Steroid biosynthesis pathway, 7-Dehydrocholesterol was linked to 28 gut microbiotas, including *Janthinobacterium*, *Aeromonas*, and *Microtrichales*. This indicates complex interplay between gut microbiota and metabolites, suggesting that gut microbiotas may indirectly influence joint health by modulating metabolite levels, providing a new perspective for the study of the ‘‘gut-joint’’ axis. These connections may be crucial to the therapeutic mechanisms of EA in treating OA. For example, specific gut microbiotas may influence metabolite levels through their metabolic activities, and these metabolites, in turn, can feedback-regulate gut microbiota growth and function. In summary, our research offers novel insights into the role of the “gut-joint” axis in OA, highlighting that the interplay between gut microbiota and metabolites may be pivotal in the disease progression.

This study still has several limitations. It was conducted in an animal model, and clinical validation is still needed. The sample size was relatively small, especially for 16S rRNA sequencing and metabolomics analyses, which may not have fully captured inter-individual variability. In addition, no sham acupuncture group was included. As a result, the specific effects of EA could not be completely separated from non-specific effects related to handling or restraint stress. Although EA treatment was associated with changes in gut microbiota and metabolites, the present data support an association rather than causation. Further studies are needed to clarify the underlying mechanisms.

## Conclusion

This research provided experimental evidence for the use of EA in OA and suggested that its therapeutic effects may involve multiple mechanisms. We found that EA was associated with changes in gut microbiota abundance and metabolites such as 7-Dehydrocholesterol in the steroid biosynthesis pathway, which may contribute to the improvement of OA. These findings provide scientific support for the application of EA as a non-pharmacological therapeutic modality and may also offer new directions for future treatment strategies for OA.

## Data Availability

The original contributions presented in the study are publicly available. This data can be found here: http://www.ncbi.nlm.nih.gov/bioproject/1499411.

## References

[B1] Basak S. Hridayanka K. S. N. Duttaroy A. K. (2023). Bioactives and their roles in bone metabolism of osteoarthritis: evidence and mechanisms on gut-bone axis. Front. Immunol. 14, 1323233. 10.3389/fimmu.2023.1323233 38235147 PMC10792057

[B2] Cao F. Xu Z. Li X. X. Fu Z. Y. Han R. Y. Zhang J. L. (2024). Trends and cross-country inequalities in the global burden of osteoarthritis, 1990-2019: a population-based study. Ageing Res. Rev. 99, 102382. 10.1016/j.arr.2024.102382 38917934

[B3] Chandrasekaran P. Weiskirchen S. Weiskirchen R. (2024). Effects of probiotics on gut microbiota: an overview. Int. J. Mol. Sci. 25 (11), 6022. 10.3390/ijms25116022 38892208 PMC11172883

[B4] Chen Y. Li M. Guo K. (2024). Exploring the mechanisms and current status of acupuncture in alleviating tumor metabolism and associated diseases: insights from the central nervous system and immune microenvironment. SLAS Technol. 29 (6), 100208. 10.1016/j.slast.2024.100208 39396727

[B5] Domingo-Almenara X. Siuzdak G. (2020). Metabolomics data processing using XCMS. Methods Mol. Biol. 2104, 11–24. 10.1007/978-1-0716-0239-3_2 31953810

[B6] Erasmus S. Lyu Z. Zhou J. Fang J. Liang Y. (2024). Electroacupuncture mechanisms in managing preoperative anxiety and postoperative pain chronification: a review. J. Pain Res. 17, 4089–4100. 10.2147/JPR.S498373 39650212 PMC11625428

[B7] Gaspar M. G. Núñez-Carro C. Blanco-Blanco M. Blanco F. J. de Andrés M. C. (2025). Inflammaging contributes to osteoarthritis development and human microbiota variations and *vice versa:* a systematic review. Osteoarthr. Cartil. 33 (2), 218–230. 10.1016/j.joca.2024.11.005 39612977

[B8] Guan Z. Luo L. Liu S. Guan Z. Zhang Q. Li X. (2022). The role of depletion of gut microbiota in osteoporosis and osteoarthritis: a narrative review. Front. Endocrinol. (Lausanne) 13, 847401. 10.3389/fendo.2022.847401 35418947 PMC8996773

[B9] He Y. Jiang W. Wang W. (2024). Global burden of osteoarthritis in adults aged 30 to 44 years, 1990 to 2019: results from the global burden of disease study 2019. BMC Musculoskelet. Disord. 25 (1), 303. 10.1186/s12891-024-07442-w 38641788 PMC11027234

[B10] Keller R. K. Arnold T. P. Fliesler S. J. (2004). Formation of 7-dehydrocholesterol-containing membrane rafts *in vitro* and *in vivo*, with relevance to the Smith-Lemli-Opitz syndrome. J. Lipid Res. 45 (2), 347–355. 10.1194/jlr.M300232-JLR200 14594996 PMC2851617

[B11] Kloppenburg M. Namane M. Cicuttini F. (2025). Osteoarthr. Lancet 405 (10472), 71–85. 10.1016/S0140-6736(24)02322-5 39755397

[B12] Li B. Jing L. Jia L. Qian T. Jianyi C. Zhongsheng H. (2020). Acupuncture reduces pain in rats with osteoarthritis by inhibiting MCP2/CCR2 signaling pathway. Exp. Biol. Med. (Maywood) 245 (18), 1722–1731. 10.1177/1535370220952342 32878462 PMC7802388

[B13] Li T. Ma X. Wang T. Tian W. Liu J. Shen W. (2024a). Clostridium butyricum inhibits the inflammation in children with primary nephrotic syndrome by regulating Th17/Tregs balance *via* gut-kidney axis. BMC Microbiol. 24 (1), 97. 10.1186/s12866-024-03242-3 38521894 PMC10960420

[B14] Li Y. Ran Q. Duan Q. Jin J. Wang Y. Yu L. (2024b). 7-Dehydrocholesterol dictates ferroptosis sensitivity. Nature 626 (7998), 411–418. 10.1038/s41586-023-06983-9 38297130 PMC11298758

[B15] Liu R. Xu N. G. Yi W. Ji C. (2020). Electroacupuncture attenuates inflammation after ischemic stroke by inhibiting NF-κB-Mediated activation of microglia. Evid. Based Complement. Altern. Med. 2020, 8163052. 10.1155/2020/8163052 32922507 PMC7453260

[B16] Martin C. S. Crastin A. Sagmeister M. S. Kalirai M. S. Turner J. D. MacDonald L. (2024). Inflammation dynamically regulates steroid hormone metabolism and action within macrophages in rheumatoid arthritis. J. Autoimmun. 147, 103263. 10.1016/j.jaut.2024.103263 38851089

[B17] Midya S. Banerjee A. Pathak S. Duttaroy A. K. (2024). “Gut microbiota and its importance in health and disease,” in Microbiota and dietary mediators in Colon cancer prevention and treatment. Editors Pathak S. Banerjee A. Duttaroy A. K. (Singapore: Springer Nature Singapore), 1–19.

[B18] Pang Z. Lu Y. Zhou G. Hui F. Xu L. Viau C. (2024). MetaboAnalyst 6.0: towards a unified platform for metabolomics data processing, analysis and interpretation. Nucleic Acids Res. 52 (W1), W398–w406. 10.1093/nar/gkae253 38587201 PMC11223798

[B19] Philpott H. T. McDougall J. J. (2020). Combatting joint pain and inflammation by dual inhibition of monoacylglycerol lipase and cyclooxygenase-2 in a rat model of osteoarthritis. Arthritis Res. Ther. 22 (1), 9. 10.1186/s13075-020-2096-3 31937359 PMC6961325

[B20] Le Pogam P. Le Page Y. Habauzit D. Doué M. Zhadobov M. Sauleau R. (2019). Untargeted metabolomics unveil alterations of biomembranes permeability in human HaCaT keratinocytes upon 60 GHz millimeter-wave exposure. Sci. Rep. 9 (1), 9343. 10.1038/s41598-019-45662-6 31249327 PMC6597695

[B21] Prosty C. Katergi K. Papenburg J. Lawandi A. Lee T. C. Shi H. (2024). Causal role of the gut microbiome in certain human diseases: a narrative review. eGastroenterology 2 (3), e100086. 10.1136/egastro-2024-100086 39944364 PMC11770457

[B22] Pu W. Liu Q. Xue S. Li S. Nan N. Liu Y. (2026). Time- and dose-related pathological changes in knee osteoarthritis rat model induced by monosodium iodoacetate. Anim. Model Exp. Med. 9 (1), 21–30. 10.1002/ame2.70037 40501372 PMC12907984

[B23] Rasmussen J. A. Villumsen K. R. Ernst M. Hansen M. Forberg T. Gopalakrishnan S. (2022). A multi-omics approach unravels metagenomic and metabolic alterations of a probiotic and synbiotic additive in rainbow trout (*Oncorhynchus mykiss*). Microbiome 10 (1), 21. 10.1186/s40168-021-01221-8 35094708 PMC8802455

[B24] De Roover A. Escribano-Núñez A. Monteagudo S. Lories R. (2023). Fundamentals of osteoarthritis: inflammatory mediators in osteoarthritis. Osteoarthr. Cartil. 31 (10), 1303–1311. 10.1016/j.joca.2023.06.005 37353140

[B25] Siddiq M. A. B. Oo W. M. Hunter D. J. (2024). New therapeutic strategies in osteoarthritis. Jt. Bone Spine 91 (6), 105739. 10.1016/j.jbspin.2024.105739 38685527

[B26] de Sire A. Mancuso E. Marotta N. Massimino M. Zito R. Averta C. (2025). Association between gut microbiota composition and physical functioning in patients with knee osteoarthritis: a machine learning study. Sci. Rep. 15 (1), 40826. 10.1038/s41598-025-24500-y 41258193 PMC12630774

[B27] Song B. Hao M. Zhang S. Niu W. Li Y. Chen Q. (2024). Comprehensive review of Hesperetin: advancements in pharmacokinetics, pharmacological effects, and novel formulations. Fitoterapia 179, 106206. 10.1016/j.fitote.2024.106206 39255908

[B28] Sun D. Bai R. Zhou W. Yao Z. Liu Y. Tang S. (2021). Angiogenin maintains gut microbe homeostasis by balancing α-Proteobacteria and Lachnospiraceae. Gut 70 (4), 666–676. 10.1136/gutjnl-2019-320135 32843357 PMC7904960

[B29] Trinh K. Zhou F. Belski N. Deng J. Wong C. Y. (2022). The effect of acupuncture on hand and wrist pain intensity, functional status, and quality of life in adults: a systematic review. Med. Acupunct. 34 (1), 34–48. 10.1089/acu.2021.0046 35251436 PMC8886934

[B30] Vo D.-K. Trinh K. T. L. (2024). Emerging biomarkers in metabolomics: advancements in precision health and disease diagnosis. Int. J. Mol. Sci. 25 (23), 13190. 10.3390/ijms252313190 39684900 PMC11642057

[B31] Wan K. Xu Q. Shi Y. Cui C. Lei J. Zhang K. (2025). Electroacupuncture produces analgesic effects *via* cannabinoid CB1 receptor-mediated GABAergic neuronal inhibition in the rostral ventromedial medulla. Chin. Med. 20 (1), 30. 10.1186/s13020-025-01083-4 40038719 PMC11881457

[B32] Wang M. Liu L. Zhang C. S. Liao Z. Jing X. Fishers M. (2020). Mechanism of traditional Chinese medicine in treating knee osteoarthritis. J. Pain Res. 13, 1421–1429. 10.2147/JPR.S247827 32606908 PMC7304682

[B33] Wang H. F. Chen L. Xie Y. Wang X. F. Yang K. Ning Y. (2021). Electroacupuncture facilitates M2 macrophage polarization and its potential role in the regulation of inflammatory response. Biomed. Pharmacother. 140, 111655. 10.1016/j.biopha.2021.111655 34029955

[B34] Wang N. Yang J. Gan G. Bao X. Wang L. (2022). Self-assembled insulin-like growth factor 1 peptides induce adipose stem cell differentiation to repair cartilage injury. Biomater. Adv. 137, 212845. 10.1016/j.bioadv.2022.212845 35929274

[B35] Wang M. Liu W. Ge J. Liu S. (2023). The immunomodulatory mechanisms for acupuncture practice. Front. Immunol. 14, 1147718. 10.3389/fimmu.2023.1147718 37090714 PMC10117649

[B36] Wang Y. Zeng T. Tang D. Cui H. Wan Y. Tang H. (2025). Integrated multi-omics analyses reveal lipid metabolic signature in osteoarthritis. J. Mol. Biol. 437 (6), 168888. 10.1016/j.jmb.2024.168888 39643156

[B37] Wen C. Xu L. Xu X. Wang D. Liang Y. Duan L. (2021). Insulin-like growth factor-1 in articular cartilage repair for osteoarthritis treatment. Arthritis Res. and Ther. 23 (1), 277. 10.1186/s13075-021-02662-0 34717735 PMC8556920

[B38] Wishart D. S. (2019). Metabolomics for investigating physiological and pathophysiological processes. Physiol. Rev. 99 (4), 1819–1875. 10.1152/physrev.00035.2018 31434538

[B39] Wu Z. Yu X. Xiong J. Wu G. Zuo Z. Xie Q. (2020). Acupuncture and moxibustion therapy for scapulohumeral periarthritis: protocol for an overview of systematic reviews and meta-analysis. Med. Baltim. 99 (35), e21567. 10.1097/MD.0000000000021567 32871872 PMC7458254

[B40] Xi Y. Wang Z. Wei Y. Xiao N. Duan L. Zhao T. (2025). Gut microbiota and osteoarthritis: from pathogenesis to novel therapeutic opportunities. Am. J. Chin. Med. 53 (1), 43–66. 10.1142/S0192415X2550003X 39880660

[B41] Xu Z.-F. Hong S.-h. Wang S. Zhao X.-y. Liu Y. Ding S. (2020). Neuroendocrine-immune regulating mechanisms for the anti-inflammatory and analgesic actions of acupuncture. World J. Traditional Chin. Med. 6 (4), 384–392. 10.4103/wjtcm.wjtcm_41_20

[B42] Yin S. Chang Y. Yan X. Feng X. Wu N. (2023). Effect of acupuncture for patients with knee osteoarthritis: study protocol for a double-dummy randomized controlled trial. J. Orthop. Surg. Res. 18 (1), 779. 10.1186/s13018-023-04198-2 37848962 PMC10583394

[B43] Yu F. Zhu C. Wu W. (2025). Senile osteoarthritis regulated by the gut microbiota: from mechanisms to treatments. Int. J. Mol. Sci. 26 (4), 1505. 10.3390/ijms26041505 40003971 PMC11855920

[B44] Zaplana T. Miele S. Tolonen A. C. (2023). Lachnospiraceae are emerging industrial biocatalysts and biotherapeutics. Front. Bioeng. Biotechnol. 11, 1324396. 10.3389/fbioe.2023.1324396 38239921 PMC10794557

[B45] Zhang W. Zhang L. Yang S. Wen B. Chen J. Chang J. (2023). Electroacupuncture ameliorates knee osteoarthritis in rats *via* inhibiting NLRP3 inflammasome and reducing pyroptosis. Mol. Pain 19, 17448069221147792. 10.1177/17448069221147792 36510338 PMC9841849

[B46] Zhu S. Zhong S. Cheng K. Zhang L. S. Bai J. W. Cao Z. (2023). Vitamin B6 regulates IL-33 homeostasis to alleviate type 2 inflammation. Cell. and Mol. Immunol. 20 (7), 794–807. 10.1038/s41423-023-01029-6 37217797 PMC10310729

[B47] Zhu D. Wang X. Xi Z. Chen K. Feng Y. Zi C. (2024a). Diet influences knee osteoarthritis osteophyte formation *via* gut microbiota and serum metabolites. iScience 27 (6), 110111. 10.1016/j.isci.2024.110111 38957790 PMC11217616

[B48] Zhu Y. Chen B. Zhang X. Akbar M. T. Wu T. Zhang Y. (2024b). Exploration of the muribaculaceae family in the gut microbiota: diversity, metabolism, and function. Nutrients 16 (16), 2660. 10.3390/nu16162660 39203797 PMC11356848

